# Integrative Analysis of Gene Expression and DNA Methylation Depicting the Impact of Obesity on Breast Cancer

**DOI:** 10.3389/fcell.2022.818082

**Published:** 2022-03-08

**Authors:** Zhenchong Xiong, Xing Li, Lin Yang, Linyu WU, Yi Xie, Fei Xu, Xinhua Xie

**Affiliations:** ^1^ Department of Breast Oncology, Sun Yat-sen University Cancer Center, State Key Laboratory of Oncology in South China, Collaborative Innovation Center of Cancer Medicine, Guangzhou, China; ^2^ Department of Radiation Oncology, Nanfang Hospital, Southern Medical University, Guangzhou, China

**Keywords:** obesity, breast cancer, immune checkpoint inhibitor, DNA methylation, biomarker

## Abstract

Obesity has been reported to be a risk factor for breast cancer, but how obesity affects breast cancer (BC) remains unclear. Although body mass index (BMI) is the most commonly used reference for obesity, it is insufficient to evaluate the obesity-related pathophysiological changes in breast tissue. The purpose of this study is to establish a DNA-methylation-based biomarker for BMI (DM-BMI) and explore the connection between obesity and BC. Using DNA methylation data from The Cancer Genome Atlas (TCGA) and Gene Expression Omnibus (GEO), we developed DM-BMI to evaluate the degree of obesity in breast tissues. In tissues from non-BC and BC population, the DM-BMI model exhibited high accuracy in BMI prediction. In BC tissues, DM-BMI correlated with increased adipose tissue content and BC tissues with increased DM-BMI exhibited higher expression of pro-inflammatory adipokines. Next, we identified the gene expression profile relating to DM-BMI. Using Gene Ontology (GO) and the Kyoto Encyclopedia of Genes and Genomes (KEGG) database, we observed that the DM-BMI-related genes were mostly involved in the process of cancer immunity. DM-BMI is positively correlated with T cell infiltration in BC tissues. Furthermore, we observed that DM-BMI was positively correlated with immune checkpoint inhibitors (ICI) response markers in BC. Collectively, we developed a new biomarker for obesity and discovered that BC tissues from obese individuals exhibit an increased degree of immune cell infiltration, indicating that obese BC patients might be the potential beneficiaries for ICI treatment.

## Introduction

Breast cancer (BC) is the most commonly diagnosed cancer and the second leading cause of cancer death for women in the world (Bray et al., 2018). It was reported in previous studies that obesity, characterized by excess adipose tissues, is a risk factor for BC (Pierobon and Frankenfeld, 2013; Sung et al., 2019). For premenopausal women, obesity is connected with increased risk of hormone receptor (HR) negative BC, while for postmenopausal women, it is connected with increased risk of HR positive BC (Suzuki et al., 2009; Picon-Ruiz et al., 2017). Moreover, several studies showed that obese patients exhibited more aggressive tumor features (such as larger tumor size, lymph node metastasis, shorter disease-free survival, and greater risk of mortality) compared with non-obese patients in BC (Copson et al., 2015; Jiralerspong and Goodwin, 2016). Although it has been observed in previous studies that the adipose tissue in obese individuals increasingly secrets adipokines (including hormones, growth factors, and cytokines) and contributes to an environment promoting cancer proliferation and metastasis (Khandekar et al., 2011; Maguire et al., 2021), how obesity impacts BC requires further studies.

Body mass index (BMI), defined as a person’s weight in kilograms divided by the square of height in meters, is the most commonly used method for obesity evaluation. However, it is more like a surrogate measure for body fatness for obesity should be calculated using the excess accumulation of adipose tissues rather than body mass (Prentice and Jebb, 2001). As there is heterogeneity in the body distribution, function, and tissue composition of adipose tissue among BC patients, a total body mass index is insufficient to evaluate the degree of obesity in local tissue. Moreover, BMI is only able to reflect the gaining of weight, with no indication in pathophysiological changes during the process of obesity (Bosello et al., 2016). Thus, developing new biomarkers to evaluate the obesity status of BC tissues is helpful to assess the impact of obesity on BC.

It is well known that obesity is affected by multiple factors (including environmental factors, genetic predisposition, and the individual lifestyle) (Conway and Rene, 2004; Bray et al., 2016). Recently, increased evidences showed that DNA methylation is also involved in the process of obesity (Ling and Rönn, 2019; Samblas et al., 2019). DNA methylation is an epigenetic mechanism which regulates gene expression through chromatin structure changes. Equally influenced by environmental factors, genetic predisposition and the individual lifestyle, the level of gene methylation is dynamically changing in setting up stable gene expression profiles to adapt to the process of obesity (Samblas et al., 2019; Cabre et al., 2021). A previous study analyzing the whole genome methylation and gene expression in non-diseased breast showed that obesity is connected with the genome-wide methylation changes in human tissue (Hair et al., 2015a). In addition, Hair et al. observed that obesity is significantly correlated with genome-wide hyper-methylation in ER-positive BCs (Hair et al., 2015b). Thus, changes of genome-wide DNA methylation could be a reflection of the biological changes in breast tissue during the process of obesity. The goal of our study is to capture the obesity-related genomic changes and explore the impact of obesity on BC tissues. We developed DNA methylation-based BMI index (DM-BMI) to evaluate the degree of obesity in breast tissues and validated the accuracy of DM-BMI in breast tissues from non-BC and BC population. Furthermore, we assessed the correlation among DM-BMI, obesity adipose tissue content, and the expression of adipokines in BC tissues. Next, we identified the DM-BMI-related gene expression profile. Using Gene Ontology (GO) and Kyoto Encyclopedia of Genes and Genomes (KEGG) database, we observed that the DM-BMI-related genes are significantly involved in the process of cancer immunity. Using Estimate and Cibersort algorithm, we observed a positive correlation between DM-BMI and immune cell infiltration. Finally, we assessed the correlation between DM-BMI and biomarkers of response to immune checkpoint inhibitors (ICI) (Shah et al., 2012) and observed that DM-BMI positively correlated with ICI response in BC.

## Materials and methods

### Data collection and processing

The training set includes genome-wide methylation data of 221 normal breast tissues in GEO (GSE88883 and GSE101961) while the validation sets includes data of 44 normal breast tissues (Validation Set 1). Data of 70 tumor-adjacent breast tissues (Validation Set 2) in GEO (GSE67919 and GSE74214) were used to develop the DM-BMI score. BMI data of the above cases are listed in Supplementary Materials S1, S2. The DNA methylation and expression data of 76 cases with matched tumor and tumor-adjacent breast tissues and the data of 775 cases with unmatched tumor tissues were collected from The Cancer Genome Atlas (TCGA) database (https://portal.gdc.cancer.gov/). Clinical features of BC patients in TCGA-BRCA are listed in Table 1.

**TABLE 1:** Clinicopathological features for TCGA-BRCA cases

	Unmatched BC cases (n = 775)	Matched BC cases (n = 76)
ER status (%)		
+	155 (20%)	12 (15.8%)
−	512 (66.1%)	53 (69.7%)
Unknown	108 (13.9%)	11 (14.5%)
PR status (%)		
+	216 (27.9%)	19 (25%)
**-**	449 (57.9%)	45 (59.2%)
Unknown	110 (14.2%)	12 (15.8%)
HER2 status (%)		
+	469 (60.5%)	41 (53.9%)
-	93 (12%)	12 (15.8%)
Unknown	213 (27.5%)	23 (30.3%)
T stage (%)		
T1	199 (25.7%)	17 (22.4%)
T2	442 (57%)	46 (60.5%)
T3	108 (13.9%)	8 (10.5%)
T4	23 (3%)	5 (6.6%)
Unknown	3 (0.4%)	0 (0%)
N stage (%)		
N1	346 (44.6%)	26 (34.3%)
N2	268 (34.6%)	34 (44.7%)
N3	95 (12.3%)	9 (11.8%)
N4	55 (7.1%)	4 (5.3%)
Unknown	11 (11.4%)	3 (3.9%)
M stage (%)		
M0	610 (78.7%)	70 (92.1%)
M1	13 (1.7%)	1 (1.3%)
MX	152 (19.6%)	5 (6.6%)
Molecular subtype (%)		
Normal-like	33 (4.3%)	1 (1.3%)
Luminal A	370 (47.7%)	41 (53.9%)
Luminal B	120 (15.5%)	20 (26.4%)
Her-2	39 (5%)	6 (7.9%)
TNBC	125 (16.1%)	8 (10.5%)
Unknown	88 (11.4%)	0 (0%)
Vital status (%)		
Alive	672 (86.7%)	45 (59.2%)
Death	103 (13.3%)	31 (40.8%)

**Abbreviation**: ER = Estrogen receptor; PR = Progesterone receptor

Genome-wide methylation data was profiled using Illumina Infinium HumanMethylation450 BeadChips Assay. For DNA methylation data, *β* value ranging from 0 (no DNA methylation) to 1 (complete DNA methylation) was used to define the methylation level of each probe. Probes with missing value in over 50% samples were removed while the probes with missing value in less than 50% of samples were imputed with the k-nearest neighbors (knn) imputation method. Probes located on the sex chromosome and probes containing known single nucleotide polymorphisms (SNPs) were removed. Eventually, 301,998 probes were included in this study. BMIQ normalization for type I and II probe correction was performed. Data from multiple studies was integrated and the Combat algorithm was performed to remove the batch effects. The above processes were carried out using the R package ChAMP (Tian et al., 2017).

For gene expression data, background correction and normalization were carried out using the R package limma (Ritchie et al., 2015).

### Calculation of DNA-methylation based body mass index

To improve the predictive accuracy of the model, the BMI value was transformed to F(BMI) before further analysis, which is shown as follows:

F(BMI)=log(BMI+1)-log(healthy.BMI+1) if BMI<=healthy.BMI (1)

F(BMI)=(BMI-healthy.BMI)/(healthy.BMI +1) if BMI>healthy.BMI (2)

The parameter healthy.BMI was set to 25, referring to the upper limit of BMI in healthy population.

A lasso regression was used to regress the DM-BMI in the form of F(BMI) based on the BMI data and DNA methylation data with 301,998 probes; 42 probes were selected in the lasso regression model as BMI predictors according to the lambda.min value (Supplementary Figure S1A). The coefficient values of each probe are shown in Supplementary Figure S1B. The lasso regression analysis was carried out using R package glmnet (alpha was set to 1, and the lambda value was identified by performing a 10-fold cross validation to the training data).

### Analysis of intra-sample adipose tissue content

Adipose tissue accounts for a large proportion of breast tissue composition. Based on DNA methylation, we used a deconvolution algorithm to calculate the proportion of adipose tissue in breast and BC tissues. Teschendorff et al. (2016) provided a deconvolution algorithm to model cell subpopulations in breast tissues based on DNA methylation data. Illumina 450k DNA methylation data of human mammary epithelial cells (GSE40699) and adipose tissue (GSE48472) were used as reference profiles. Data were processed as previously described. Probes with an absolute difference in beta-value between the human mammary epithelial cells and the averaged adipose tissue >0.7 were selected for the evaluation of intra-sample adipose tissue content. Data for adipose tissue content are listed in Supplementary Material S3.

### Characteristics analyses of body mass index predictors

Forty-two probes were selected in the lasso regression model as BMI predictors. The distribution of 42 probes on chromosome, CpG island, and TSS regions was assessed using the R package ChAMP. BMI predictors, differentially methylated between BC tissues and tumor-adjacent breast tissues in the TCGA database, were identified using the R package Champ. The survival correlation of BMI predictors was assessed using TCGA BRCA data. Correlation between the methylation level of BMI predictors and DM-BMI was assessed.

### Functional and clinical characteristics analysis of DM-BMI-related gene profile

DM-BMI of TCGA-BC tissues were calculated using DNA methylation data. Spearman correlation coefficient was used to assess the correlation of DM-BMI and clinical characteristics (menopause status, hormone status, copy number variation and gene mutation) in BC. Gene expression profile related to DM-BMI was identified; functional analysis of the related genes was process by GO and KEGG analysis. Besides, we performed gene set variation analysis (GSVA) to identify DM-BMI related gene signature using gene expression data in TCGA (Hänzelmann et al., 2013). The above procedures were performed using the R software.

### Evaluation of correlations between DM-BMI and the immune microenvironment in breast cancer

Tumor mutation burden (TMB) was defined as the number of non-synonymous mutations/Mb of genome. As previously reported (Chalmers et al., 2017), TMB of BC tissues in TCGA was calculated as (whole exome non-synonymous mutations)/38 (Mb).

The level of tumor-infiltrating immune cells and stromal cells in each tissue were evaluated by ESTIMATE algorithm (Yoshihara et al., 2013). The proportion of 22 immune cells in each tissue was evaluated using the CIBERSORT algorithm (http://cibersort.stanford.edu/) (Gentles et al., 2015). Correlations between DM-BMI and ESTIMATE/CIBERSORT scores were calculated using Spearman correlation coefficient. The data for TMB, ESTIMATE, and CIBERSORT analysis are listed in Supplementary Material S4.

### Evaluation of correlations between DM-BMI and the cancer immunotherapy response

Biomarkers used to predict the immunotherapy response includes: IFN-γ signature (IFNG) (Ayers et al., 2017), IFNG hallmark gene set (IFNG.GS) (Benci et al., 2019), antigen processing and presenting machinery (APM) score (Leone et al., 2013), CD274, CD8, Tumour Immune Dysfunction and Exclusion (TIDE) (Jiang et al., 2018), myeloid-derived suppressor cells (MDSCs), cancer-associated fibroblasts (CAFs), and the M2 subtype of tumor-associated macrophages (TAM-M2) (Joyce and Fearon, 2015). IFNG was calculated by averaging six genes (IFNG, STAT1, IDO1, CXCL9, CXCL10, HLA-DRA) (Ayers et al., 2017). IFNG.GS was calculated as the average expression of all genes in the set (Benci et al., 2019). APS was defined as the mRNA expression status of APM genes (PSMB5, PSMB6, PSMB7, PSMB8, PSMB9, PSMB10, TAP1, TAP2, ERAP1, ERAP2, CANX, CALR, PDIA3, TAPBP, B2M, HLA-A, HLA-B, and HLA-C) (Leone et al., 2013). CD274, CD8, TIDE, MDSCs, CAFs, and TAM-M2 were calculated using the web application (http://tide.dfci.harvard.edu). The relevant data are listed in Supplementary Material S4.

## Results

### Development and validation of DM-BMI in breast, tumor-adjacent, and breast cancer tissues

**FIGURE 1:**
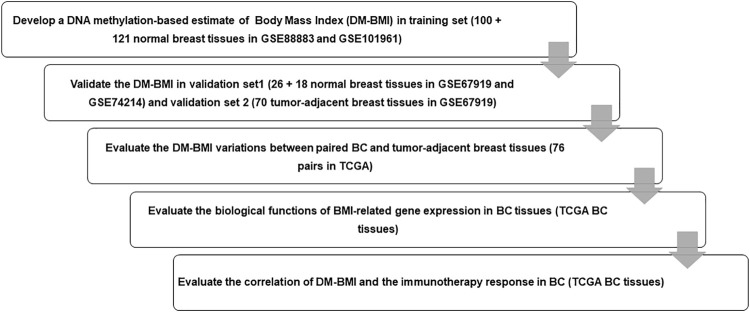
Flow chart of the study design. We enrolled 221 normal breast tissues as training set to develop a lasso regression to predict DM-BMI and validated the accuracy of the model with data from normal breast tissues and tumor-adjacent breast tissues. Then, we predicted DM-BMI in 775 BC tissues and 76 matched tumor-adjacent breast tissues. The correlation between DM-BMI and clinical characteristics was assessed in BC tissues. Further, we identified the DM-BMI related gene profile and evaluated the relationships between DN-BMI and tumor immune response in BC tissues.

**FIGURE 2:**
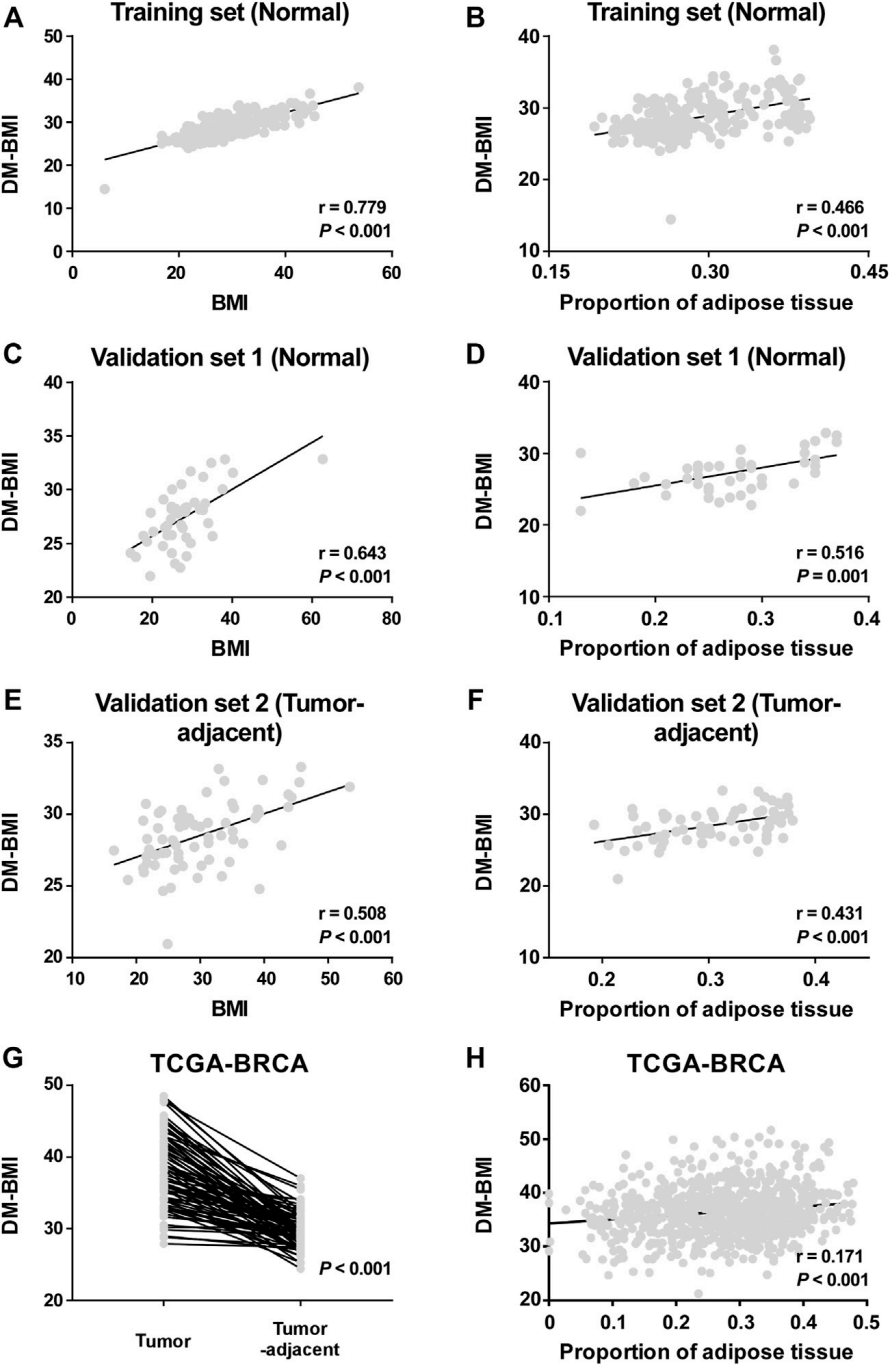
Development and validation of the DA-BMI predicting model. **(A,B)** Correlation of DM-BMI with **(A)** BMI and **(B)** proportion of adipose tissue in normal breast tissues based on the training set (GSE88883 and GSE101961). **(C,D)** Correlation of DM-BMI with **(C)** BMI and **(D)** proportion of adipose tissue in normal breast tissues based on Validation Set 1 (GSE67919 and GSE74214). **(E,F)** Correlation of DM-BMI with **(E)** BMI and **(F)** proportion of adipose tissue in tumor-adjacent breast tissues based on Validation Set 2 (GSE67919). **(G)** Analyzing the differences of DM-BMI between tumor tissues (n = 76) and matched tumor-adjacent breast tissues (n = 76) based on the TCGA-BRCA dataset. **(H)** Correlation of DM-BMI with proportion of adipose tissue in BC tissues based on the TCGA-BRCA dataset (n = 775). (A-F and H) *r*, Spearman correlation coefficient. **(G)** p-values were determined by paired *t*-test.

A total of 221 breast tissues from non-BC cases (GEO database) were selected as the training cohort to develop the DNA-methylation-based BMI (DM-BMI) prediction model (Figure 1). The median BMI and median age of the training cohort were 28.24 (6.07–53.74) and 37 (17–82). Fifty lasso regression models based on DNA methylation data of the training cohort (301,998 probes per sample) were constructed. A model with minimum mean-squared error was selected based on the Lambda value (Supplementary Figure S1A). Forty-two probes were included and their coefficients are shown in Supplementary Figure S1B and Supplementary Material S5. We used Spearman correlation coefficient and paired *t*-test to assess the predictive accuracy of the DM-BMI model. DM-BMI showed a significant correlation with BMI (Figure 2A) while paired *t*-test revealed that there was no significant difference between DM-BMI and BMI (*t* = −0.384, df = 220, p-value = 0.702). Using a deconvolution algorithm, we evaluated the breast tissue composition and observed that the increased DM-BMI was significantly correlated with higher proportion of adipose tissue (Figure 2B). These results showed the high accuracy of DM-BMI for BMI prediction.

Next, 44 normal breast cases (Validation Set 1) and 70 tumor-adjacent breast tissues (Validation Set 2) were enrolled for external validation (Figure 1). The median BMI and median age of Validation Set 1 were 27.1 (14.6–62.7) and 44 (13–80); median BMI and median age of Validation Set 2 were 28.65 (16.5–53.4) and 56 (29–84). In both Validation Sets 1 and 2, DM-BMI showed positive correlation with BMI (Figures 2C,E). Paired *t*-test revealed that there was no significant difference between DM-BMI and BMI (*t* = −0.253, *df* = 43, p-value = 0.801, Validation Set 1; *t* = −1.87, *df* = 69, p-value = 0.066, Validation Set 2). Moreover, DM-BMI is significantly correlated with adipose tissue proportion in both normal and tumor-adjacent breast tissues (Figures 2D,F). The above results showed a high prediction accuracy of DM-BMI model in normal and tumor-adjacent breast tissues.

Furthermore, we assessed the DM-BMI of paired tumor and tumor-adjacent breast tissues in TCGA database. The tumor tissues exhibited a higher level of DM-BMI compared with its paired tumor-adjacent tissues (Figure 2G). In BC tissues, DM-BMI is positively correlated with adipose tissue proportion (Figure 2H).

### What is known about the 42 body mass index predictors?

**FIGURE 3:**
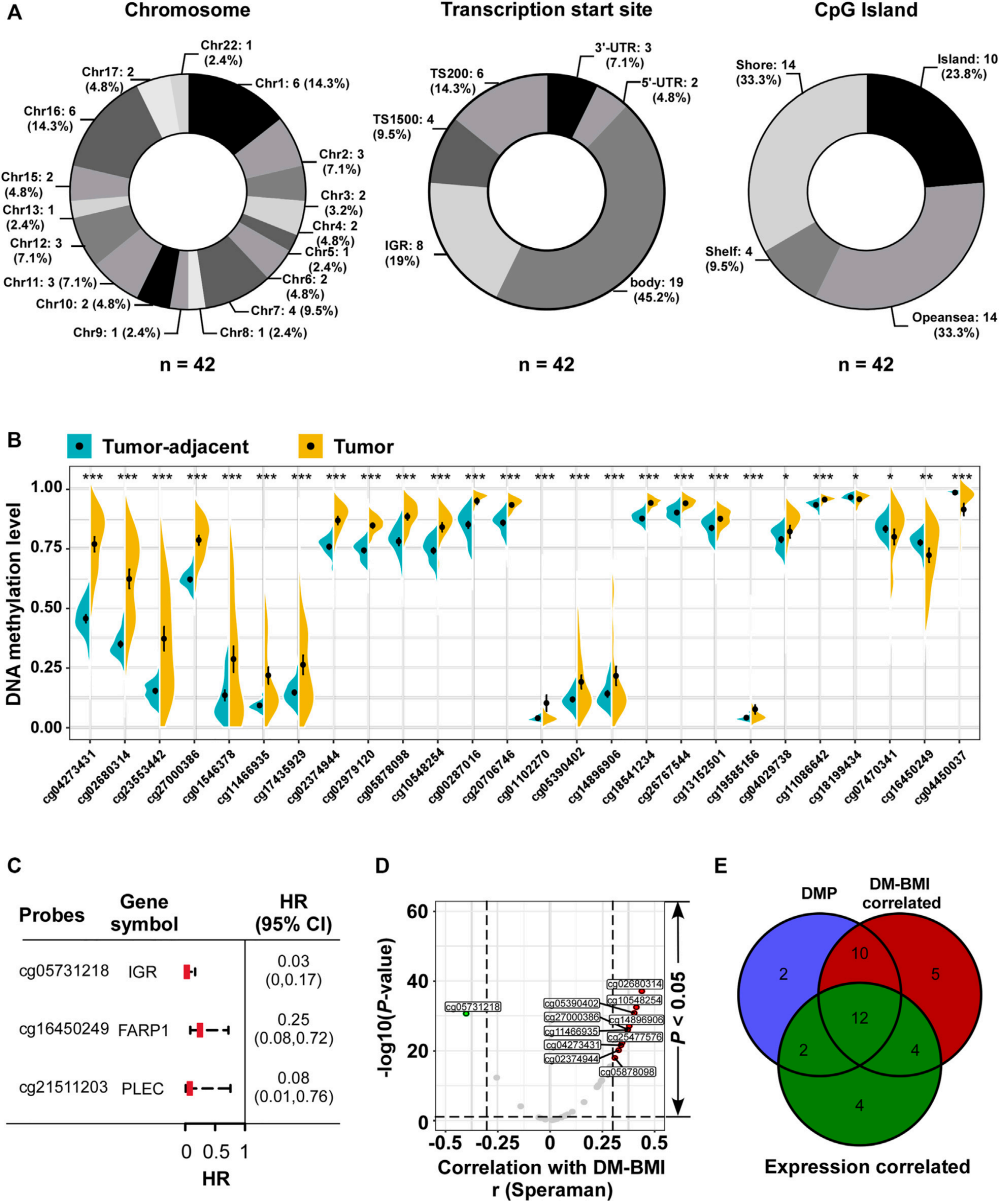
Characteristic analyses of BMI predictors. **(A)** Distribution of 42 BMI predictors referring to (Left) chromosome and (Middle) transcription Start Sites; CpG islands were listed as number (proportion). For chromosome, no BMI predictors were located in Chr14, 18, 19, 20, and 21. **(B)** Identification of differential methylation BMI predictors between tumor tissues and matched tumor-adjacent breast tissues in TCGA dataset (n = 76). *p < 0.05, **p < 0.01, ***p < 0.001. **(C)** Forest plot of the prognostic related BMI predictors referring to DNA methylation level from TCGA BC tissues (*n* = 774): three BMI predictors (mapping to FARP1, PLEC, and cg05731218 located in the intergenic region/IGR) negatively correlated with overall survival. **(D)** Volcano plot of the correlation analysis between DM-BMI and methylation level of BMI predictors. *r*, Spearman correlation coefficient; 39 BMI predictors positively correlated with DM-BMI (BMI predictors with correlation coefficient >0.3 were marked as red dot; n = 10), and five BMI predictors negatively correlated with DM-BMI (BMI predictors with Spearman correlation coefficient < −0.3 were marked as green dot; n = 1). **(E)** Venn diagram of DMP; DM-BMI-correlated and expression-correlated BMI predictors. BMI predictors differentially methylated between tumor and tumor-adjacent tissues were labeled as blue; methylation levels of the BMI predictors correlated with DM-BMI were labeled as red; methylation levels of BMI predictors negatively correlated with gene expression were labeled as green.

As DM-BMI was significantly correlated with obesity status, which has been suggested to regard as a risk factor for BC, we further assessed the relevance between BMI predictors and BC. As hyper-methylation of CpG island at gene promoter regions often causes gene silencing, we first evaluated the distribution of BMI predictors. Among 22 pairs of chromosomes (Chr), Chr1 and 16 are the most common region for BMI predictor distribution; 45.2% of BMI predictors were located at the gene body regions while only 23.8% of them were located at the promoter regions (TS1500 and TS200). Furthermore, we observed that only a few parts of BMI predictors were located at CpG islands (Figure 3A).

Next, we assessed the methylation variation of BMI predictors between tumor and tumor-adjacent breast tissues. Twenty-six differentially methylated probes (DMP) were identified: 22 BMI predictors were hyper-methylated in tumor tissues compared with the tumor-adjacent breast tissues; 4 were hypo-methylated in tumor (Figure 3B). Three of 42 BMI predictors were correlated with better OS for BC patients, while 2 of them were located at gene-coding regions (Figure 3C). In BC tissues, the correlation between the methylation level of 42 BMI predictors and DM-BMI was evaluated. Eleven of them were significantly correlated with DM-BMI (correlation coefficient >0.3 or < −0.3; Figures 3D,E); 35 of 42 BMI predictors were matched to the human gene region. Through the integrative analysis of DNA methylation and expression data, the negative correlation between methylation level and gene expression was observed in 22 of 42 BMI predictors (Figure 3E).

### Functional and clinical characteristics analysis of DM-BMI related gene profile in breast cancer

**FIGURE 4:**
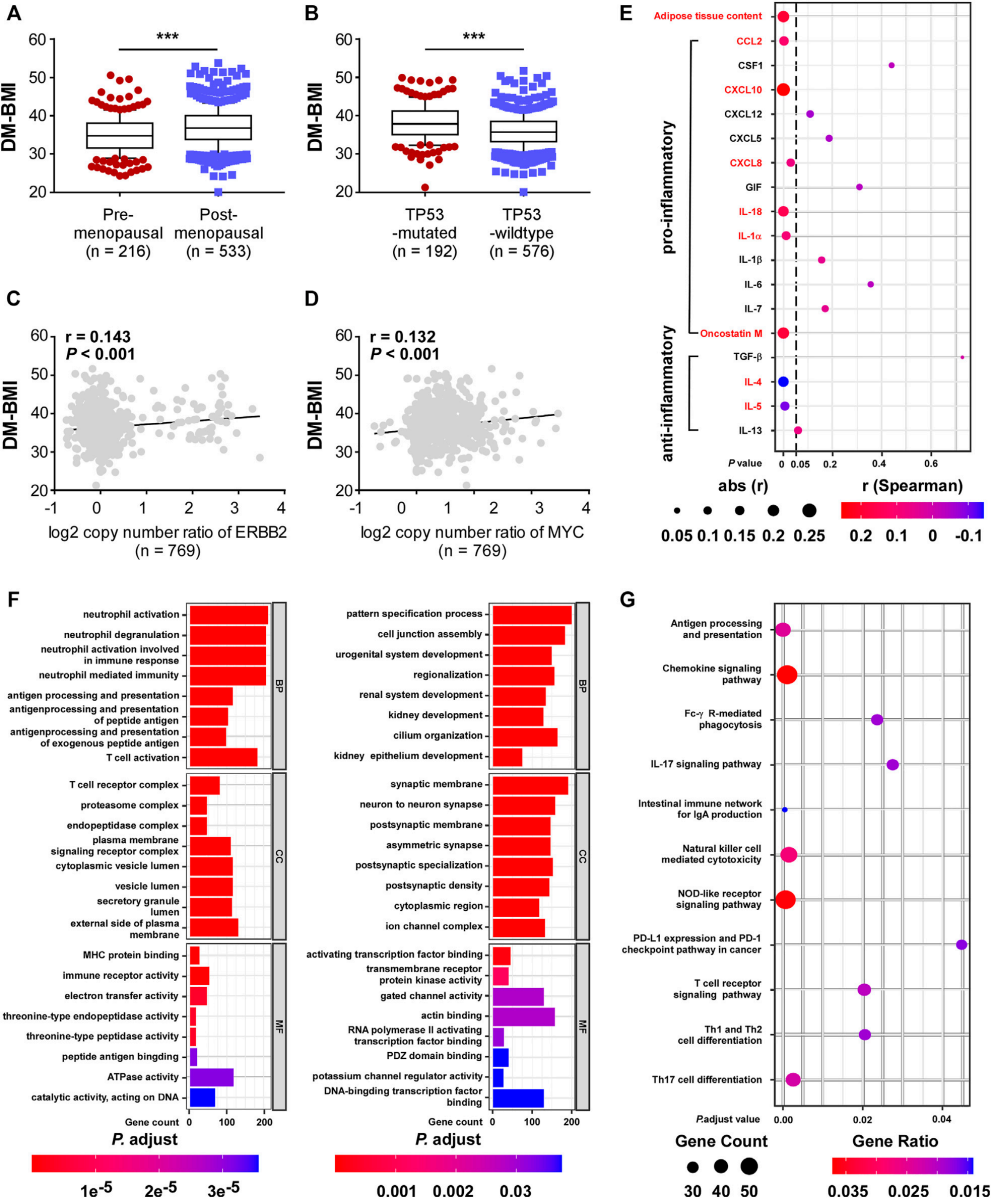
Functional and clinical characteristics analysis of DM-BMI-related gene profile in BC. **(A,B)** Analyzing the differences of DM-BMI based on **(A)** menopause status of patients (n = 749) or TP53 mutation status (n = 768) based on TCGA-BRCA dataset. **(C,D)** Correlation of DM-BMI with copy number of **(C)** ERBB2 and **(D)** MYC. *r*, Spearman correlation coefficient. **(E)** Correlation of DM-BMI and expression of pro/anti-inflammatory adipokines in BC tissues (n = 771). Expressions of adipokines significantly correlated with DM-BMI were labeled as red. **(F)** GO function analysis of DM-BMI related gene. (Left) Analysis of gene whose mRNA expression positively correlated with DM-BMI (Ayers et al., 2017); analysis of gene whose mRNA expression negatively correlated with DM-BMI. **(G)** Analysis of DM-BMI-related gene enrichment in immunologic pathway based on KEGG database. Gene ratio was defined as: number of genes enriched to target pathway/number of DM-BMI related genes included in the KEGG dataset.

Later we explored the biological significance of DM-BMI in breast cancer tissues. The survival correlation of DM-BMI was evaluated in BCs and the subgroup of BCs with cancer therapy (chemotherapy, endocrine-therapy, anti-HER2 therapy, and radiation-therapy). DM-BMI was consistently correlated with higher mortality risk in the whole cohort of BC patients and subgroups of patients with chemotherapy, endocrine-therapy or radiation-therapy, respectively (Table 2). Tissues from patients with postmenopausal status and TP53-mutation exhibited a significantly higher level of DM-BMI (Figures 4A,B). Apart from that, an increasing level of DM-BMI was correlated with an increased proportion of ERBB2 and MYC amplification (Figures 4C,D).

**TABLE 2:** Survival analysis of DM-BMI in BC with systemic therapy

Subgroup	Hazard ratio (high vs. Low)	95%CI	p-Value
Overall	1.046329459	1.005–1.089	0.027924058
Chemotherapy	1.100065801	1.029–1.176	0.00529122
Hormone therapy	1.087123098	1.009–1.171	0.027857745
Anti-HER2 therapy	1.048120675	0.832–1.320	0.689677033
Radiation therapy	1.0775886	1.003–1.158	0.041331982

Previous studies showed that adipokines produced by obese adipose tissues leads to obesity-mediated inflammation and BC progression. In BC tissues, sections of adipose tissue were positively correlated with DM-BMI. Expression of six pro-inflammatory adipokines was positively correlated with DM-BMI while the expression of two anti-inflammatory adipokines was negatively correlated with DM-BMI (Figure 4E). These results indicated that obesity increased the expression of pro-inflammatory adipokines in BC tissues.

Furthermore, we assessed the DM-BMI (obesity)-related gene expression profile and mRNA expression of 10,032 genes significantly correlated with DM-BMI. To evaluate the biological effect of obesity on BC, we performed a GSEA analysis of DM-BMI-related genes using KEGG and GO database. GO analysis showed that gens positively correlated with DM-BMI were significantly involved in antigen process and presentation, immune cell activation, MHC protein binding, and immune receptor activity in BC (Figure 4F). KEGG consistently showed that DM-BMI-related genes were significantly enriched in tumor-immunity-related pathway (which includes antigen processing and presentation, NK cell-mediated cytotoxicity, T cell differentiation, and PD-1 checkpoint pathway) (Figure 4G). These results indicated that the obesity-related gene profile is involved in the regulation of immune response in BC.

### DM-BMI correlated with T-cell infiltration and immune checkpoint inhibitor response markers in breast cancer

**FIGURE 5:**
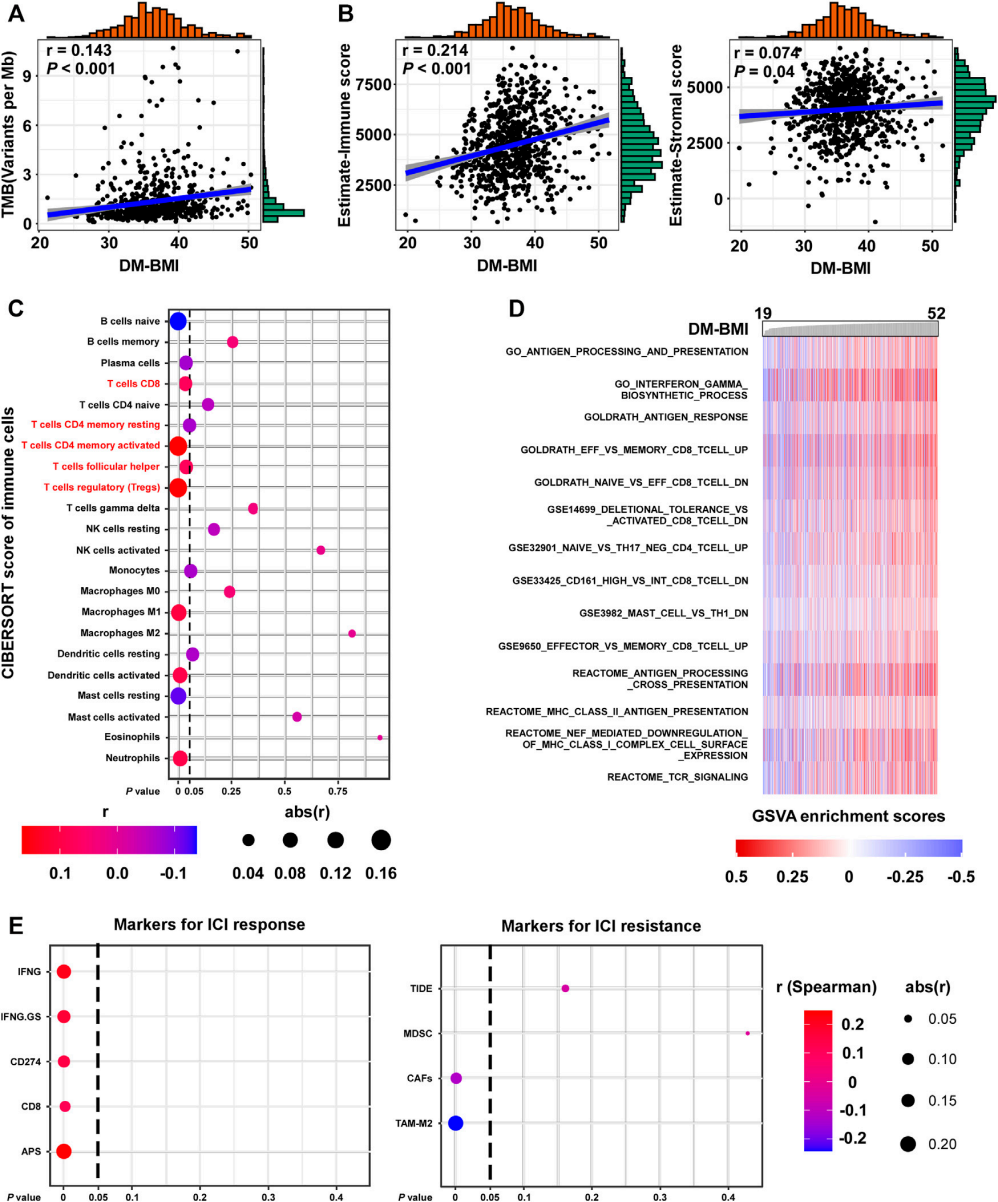
DM-BMI correlated with T cell infiltration and ICI response in BC. **(A)** Correlation of DM-BMI with tumor mutation burden (TMB) in BC tissues (TCGA-BRCA, n = 775). Numerical distribution of DM-BMI and TMB is shown on the above *x*- and the right *y*-axis, respectively. **(B)** Correlation of DM-BMI with the level of infiltrating immune cells (Left, estimate-immune score) and the level of stromal cells (Right, estimate-stromal score) in BC tissues (TCGA-BRCA, n = 772). Numerical distribution of DM-BMI and estimate-immune/stromal score is shown on the above *x*- and the right *y*-axis, respectively. **(C)** Correlation of DM-BMI with 22 types of immune cell components is shown by dotplot. Five of 7 T-cell contents correlated with DM-BMI, which were labeled in red. **(D)** GSVA analysis identified an immunologic pathway which positively correlated with DM-BMI. Enrichment scores of pathways from the GSEA-Molecular Signatures Database were calculated using GSVA in BC tissues (TCGA-BRCA-mRNA data, *n* = 771). Immunologic gene sets which significantly correlated with DM-BMI are displayed (*r* > 0.3). **(E)** Correlation of DM-BMI with markers for ICI response/resistance is shown by dotplot. For **(A,B)**, *r*, Spearman correlation coefficient.

We evaluated the correlation between obesity and immune response to BC. Gene mutation which changes the protein-coding sequence and leads to the expression of abnormal proteins was suggested to be the driving factor for cancer development. Also, the abnormal protein derived from tumor mutation might rouse immune response. In BC tissues, we observed a positive correlation between DM-BMI and TMB (Figure 5A). Using the Estimate algorithm, we evaluated the degree of immune cell infiltration in TCGA BC tissues. Interestingly, we found a positive correlation between DM-BMI and Estimate-immune score while no significant correlation was observed between DM-BMI and Estimate-Stromal score (Figure 5B). Next, we calculated the relative abundance of 22 immune cell types in TCGA BC tissues. Among them, the content of CD8-T, CD4 memory-activated T, T follicular helper, and regulatory T cells (Treg) were positively correlated with DM-BMI, indicating a more intense T cell-mediated immune response in BC tissues with increased DM-BMI (Figures 5C,D). As the representative of immunotherapy, the ICI therapy suppressed BC progression by activating T cell-mediated immune response. Thus, we examined whether DM-BMI predicted the tumor response to ICI. Five markers for ICI response and four markers for ICI resistance were selected to evaluate the tumor response. In BC tissues, DM-BMI is positively correlated with IFNG, IFNG.GS, CD274, CD8, and APS, indicating that BC tissues with increased DM-BMI exhibited a better response to ICI (Figures 5A,E). Moreover, DM-BMI was negatively correlated with two ICI resistance markers (CAFs and TAM-M2). All those results indicated that BC tissue at obesity status might exhibit a more intense response to ICI based on markers of ICI response.

## Discussion

Based on the DNA methylation pattern, we developed an obesity evaluation model (DM-BMI) in the study. We then further identified the obesity-related gene expression profile based on the DM-BMI model. Although obesity has been shown to be a BC risk factor for many years, most studies have been focusing on the correlation between obesity and clinical prognosis while studies about the biological and genomic impact of obesity on breast cancer were limited. The adipose tissue, as the major agent mediating obesity-related biological effects, is an important starting point for the study of the obesity impact. In both breast and BC tissue, we observed a positive correlation between the proportion adipose tissue content and the DM-BMI. Previous studies reported that the expansion of adipose tissue, accompanied by the dysregulation of the endocrine function (adipokine secretion) of the adipose cells, was driven by an increase in size of adipose or by a formation of a new adipose cell (Ghaben and Scherer, 2019; Quail and Dannenberg, 2019). As the DM-BMI increased, we observed an increased expression of pro-inflammatory adipokines but a decreased expression of anti-inflammatory adipokines which might synergistically induce obesity-mediated inflammation through the activation of the NF-κB pathway and a pro-oncogenic environment.

In addition to the expansion of adipose tissue, we also observed a promoting effect of obesity on immune response in BC tissue. For those obese individuals, adipose tissue expands with the increasing demand of oxygen, which induces the development of the hypoxia environment (Iwamoto et al., 2018). The activation of hypoxia signaling increases the expression of adipokines, especially the pro-inflammation adipokines (including CCL2, CXCL8, CXCL10, IL-18, IL-1α and Oncostatin M), which is involved in the recruitment of tumor-associated immune cells (Taylor and Colgan, 2017; Hou et al., 2020; McGettrick and O’Neill, 2020). Moreover, previous studies showed that adipocytes could fuel immune cells by releasing exosome-sized and lipid-filled vesicles (Flaherty et al., 2019; Zhang et al., 2020). In BC tissues, we observed that DM-BMI is positively correlated with the degree of M1 macrophages and activated dendritic cell and T cell infiltration, indicating an increase activity of tumor immune response. As T cell exhaustion is the key for tumor immune escape, previous studies have indicated that T cell exhaustion could be reversed by immune checkpoint inhibition (such as PD-1 inhibition) and replenishing activated T cells (such as CAR-T) (Bajgain et al., 2018; Bassez et al., 2021). Interestingly, in obese BC tissues, we found an increase content of CD4 +CD8 + and follicular helper T cells, which may be the result of an increased secretion of immune chemokines in adipose tissue. Although we observed a positive correlation between DM-BMI and regulatory T cell (Treg), coupled with a subset of immune cell with immunosuppressive activity, they can be interpreted as a negative feedback regulation by the immune system to maintain the stability of the immune environment after the activation of the immune response (von Boehmer and Daniel, 2013). Furthermore, our study revealed that DM-BMI is positively correlated with ICI response markers in BC tissues. These results suggest that obese BC patients may benefit from ICI. Recently, Wang et al. consistently reported that obesity is concerned with increased response of PD-1/PD-L1 blockade in an animal melanoma tumor model (Wang et al., 2019). However, as our findings were mainly supported by database analysis, data from clinical samples treated with ICI treatment are still required to validate the correlation between DM-BMI and ICI response.

With the increasing number of obese patients, the impact of obesity on the treatment of breast cancer has aroused more and more attention. We observed that the increased DM-BMI was correlated with higher mortality risk in patients with chemotherapy, hormone therapy, and radiation therapy. Although no evidence pointed out that obesity will induce drug-resistance in cancer cell, dose of chemotherapy and radiation might still be routinely reduced in obese individuals as doctors usually limit body surface area under 2 m^2^ to reduce toxicity when calculating the dose of chemotherapy (Lyman, 2012; Picon-Ruiz et al., 2017). As is highly expressed of aromatase, adipose tissue is an endocrine organ, which is an important site for estrogen biosynthesis, especially for postmenopausal women (Liedtke et al., 2012). For obese BC patients, increased expression of aromatase might cause the resistance to endocrine-therapy.

Because of the limitation of BMI in obesity evaluation, several imaging methods have been developed for obesity evaluation (including: bioimpedance analysis instruments, dual-energy x-ray absorptiometry, computed tomography, and magnetic resonance imaging) (Karlsson et al., 2013; Neamat-Allah et al., 2014). Although these new methods enabled the precise quantification of adipose tissue, the operational complexity did limit their application (Nimptsch et al., 2019). Developing new methods to evaluate the degree of obesity is of great value. Recently, an increased number of studies indicated that environmental factors (such as dietary pattern and lifestyle) will induce changes in the DNA methylation pattern predisposing to obesity and obesity and, likewise, results in genome-wide methylation variation (Wahl et al., 2017; Samblas et al., 2019). Moreover, biomarkers based on DNA methylation have been shown to be effective in obesity evaluation while most of them were only applied in blood samples (Samblas et al., 2019). Thus, we developed a DNA-methylation-based biomarker (DM-BMI) for obesity evaluation in breast tissue. In both normal breast and tumor-adjacent breast tissue, DM-BMI showed a significant correlation in both BMI and the content of adipose tissue. In addition, we also observed that DM-BMI was positively correlated with the degree of pro-inflammatory adipokine and immune cell infiltration in BC tissues. All those data indicated that DM-BMI is an effective biomarker to evaluate the biological changes in tumor tissues of obese patients.

The identification of BMI predictors naturally causes the assumption that these CpGs are critical regulators of obesity. In our study, only 11 of 42 BMI predictors were significantly correlated with DM-BMI while the others exhibited negligible correlation with DM-BMI. Although DNA methylation level was negatively correlated with gene expression in over half of BMI predictors, 45.2% of BMI predictors were located at the body region of gene sequence. How the CpGs located at the body region regulate gene expression remains unclear. As a previous study reported, the variations of DNA methylation pattern are the consequence, rather than cause, of adiposity (Wahl et al., 2017). Whether these BMI predictors are regulators of obesity or imprints of the biological process remained to be further investigated.

## Conclusion

Collectively, we established a new biomarker for obesity evaluation and discovered that BC tissues of obese individuals exhibit an increased degree of immune cell infiltration, indicating that obese BC patients might be the potential beneficiaries for ICI treatment.

## Data Availability

The datasets presented in this study can be found in online repositories. The names of the repository/repositories and accession number(s) can be found in the article/Supplementary Material.

## Abbreviations

BC, Breast Cancer; HR, Hormone Receptor; BMI, Body Mass Index; DM-BMI, DNA Methylation-Based BMI Index; TCGA, The Cancer Genome Atlas; GO, Gene Ontology; KEGG, Kyoto Encyclopedia of Genes and Genomes; ICI, Immune Checkpoint Inhibitor; GEO, Gene Expression Omnibus; knn, k-nearest neighbors; SNPs, Single Nucleotide Polymorphisms; TMB, Tumour Mutation Burden; Treg, Regulatory T Cells; IFNG, IFN-γ Signature; IFNG.GS, IFNG Hallmark Gene Set; TIDE, Tumour Immune Dysfunction and Exclusion; MDSCs, Myeloid-Derived Suppressor Cells; CAFs, Cancer-Associated Fibroblasts; TAM-M2, M2 Subtype of Tumour-Associated Macrophages; Chr, Chromosome; DMP, Differentially Methylated Probes; GSEA, Gene Set Enrichment Analysis.

## References

[B1] Ayers M. Lunceford J. Nebozhyn M. Murphy E. Loboda A. Kaufman D. R. (2017). IFN-γ-related mRNA Profile Predicts Clinical Response to PD-1 Blockade. J. Clin. Invest. 127 (8), 2930–2940. 10.1172/jci91190 28650338 PMC5531419

[B2] Bajgain P. Tawinwung S. D’Elia L. Sukumaran S. Watanabe N. Hoyos V. (2018). CAR T Cell Therapy for Breast Cancer: Harnessing the Tumor Milieu to Drive T Cell Activation. J. Immunotherapy Cancer 6 (1), 34. 10.1186/s40425-018-0347-5 PMC5944113 29747685

[B3] Bassez A. Vos H. Van Dyck L. Floris G. Arijs I. Desmedt C. (2021). A Single-Cell Map of Intratumoral Changes during Anti-PD1 Treatment of Patients with Breast Cancer. Nat. Med. 27 (5), 820–832. 10.1038/s41591-021-01323-8 33958794

[B4] Benci J. L. Johnson L. R. Choa R. Xu Y. Qiu J. Zhou Z. (2019). Opposing Functions of Interferon Coordinate Adaptive and Innate Immune Responses to Cancer Immune Checkpoint Blockade. Cell 178 (4), 933–948. e914. 10.1016/j.cell.2019.07.019 31398344 PMC6830508

[B5] Bosello O. Donataccio M. P. Cuzzolaro M. (2016). Obesity or Obesities? Controversies on the Association between Body Mass index and Premature Mortality. Eat. Weight Disordewd 21 (2), 165–174. 10.1007/s40519-016-0278-4 27043948

[B6] Bray F. Ferlay J. Soerjomataram I. Siegel R. L. Torre L. A. Jemal A. (2018). Global Cancer Statistics 2018: GLOBOCAN Estimates of Incidence and Mortality Worldwide for 36 Cancers in 185 Countries. CA: a Cancer J. clinicians 68 (6), 394–424. 10.3322/caac.21492 30207593

[B7] Bray G. A. Frühbeck G. Ryan D. H. Wilding J. P. H. (2016). Management of Obesity. The Lancet 387 (10031), 1947–1956. 10.1016/s0140-6736(16)00271-3 26868660

[B8] Cabre N. Luciano-Mateo F. Chapski D. J. Baiges-Gaya G. Fernandez-Arroyo S. Hernandez-Aguilera A. (2021). Glutaminolysis-induced mTORC1 Activation Drives Non-alcoholic Steatohepatitis Progression. J. Hepatol.. 10.1016/j.jhep.2021.04.037 33961941

[B9] Chalmers Z. R. Connelly C. F. Fabrizio D. Gay L. Ali S. M. Ennis R. (2017). Analysis of 100,000 Human Cancer Genomes Reveals the Landscape of Tumor Mutational burden. Genome Med. 9 (1), 34. 10.1186/s13073-017-0424-2 28420421 PMC5395719

[B10] Conway B. Rene A. (2004). Obesity as a Disease: No Lightweight Matter. Obes. Rev. 5 (3), 145–151. 10.1111/j.1467-789x.2004.00144.x 15245383

[B11] Copson E. R. Cutress R. I. Maishman T. Eccles B. K. Gerty S. Stanton L. (2015). Obesity and the Outcome of Young Breast Cancer Patients in the UK: the POSH Study. Ann. Oncol. 26 (1), 101–112. 10.1093/annonc/mdu509 25361993

[B12] Flaherty S. E. 3rd Grijalva A. Xu X. Ables E. Nomani A. Ferrante A. W. Jr. (2019). A Lipase-independent Pathway of Lipid Release and Immune Modulation by Adipocytes. Science 363 (6430), 989–993. 10.1126/science.aaw2586 30819964 PMC6579605

[B13] Gentles A. J. Newman A. M. Liu C. L. Bratman S. V. Feng W. Kim D. (2015). The Prognostic Landscape of Genes and Infiltrating Immune Cells across Human Cancers. Nat. Med. 21 (8), 938–945. 10.1038/nm.3909 26193342 PMC4852857

[B14] Ghaben A. L. Scherer P. E. (2019). Adipogenesis and Metabolic Health. Nat. Rev. Mol. Cel Biol 20 (4), 242–258. 10.1038/s41580-018-0093-z 30610207

[B15] Hair B. Y. Troester M. A. Edmiston S. N. Parrish E. A. Robinson W. R. Wu M. C. (2015). Body Mass index Is Associated with Gene Methylation in Estrogen Receptor-Positive Breast Tumors. Cancer Epidemiol. Biomarkers Prev. 24 (3), 580–586. 10.1158/1055-9965.epi-14-1017 25583948 PMC4355173

[B16] Hair B. Y. Xu Z. Kirk E. L. Harlid S. Sandhu R. Robinson W. R. (2015). Body Mass index Associated with Genome-wide Methylation in Breast Tissue. Breast Cancer Res. Treat. 151 (2), 453–463. 10.1007/s10549-015-3401-8 25953686 PMC4474159

[B17] Hänzelmann S. Castelo R. Guinney J. (2013). GSVA: Gene Set Variation Analysis for Microarray and RNA-Seq Data. BMC bioinformatics 14, 7. 10.1186/1471-2105-14-7 23323831 PMC3618321

[B18] Hou J. Zhao R. Xia W. Chang C.-W. You Y. Hsu J.-M. (2020). PD-L1-mediated Gasdermin C Expression Switches Apoptosis to Pyroptosis in Cancer Cells and Facilitates Tumour Necrosis. Nat. Cel Biol 22 (10), 1264–1275. 10.1038/s41556-020-0575-z PMC7653546 32929201

[B19] Iwamoto H. Abe M. Yang Y. Cui D. Seki T. Nakamura M. (2018). Cancer Lipid Metabolism Confers Antiangiogenic Drug Resistance. Cel Metab. 28 (1), 104–117. 10.1016/j.cmet.2018.05.005 29861385

[B20] Jiang P. Gu S. Pan D. Fu J. Sahu A. Hu X. (2018). Signatures of T Cell Dysfunction and Exclusion Predict Cancer Immunotherapy Response. Nat. Med. 24 (10), 1550–1558. 10.1038/s41591-018-0136-1 30127393 PMC6487502

[B21] Jiralerspong S. Goodwin P. J. (2016). Obesity and Breast Cancer Prognosis: Evidence, Challenges, and Opportunities. Jco 34 (35), 4203–4216. 10.1200/jco.2016.68.4480 27903149

[B22] Joyce J. A. Fearon D. T. (2015). T Cell Exclusion, Immune Privilege, and the Tumor Microenvironment. Science 348 (6230), 74–80. 10.1126/science.aaa6204 25838376

[B23] Karlsson A.-K. Kullberg J. Stokland E. Allvin K. Gronowitz E. Svensson P.-A. (2013). Measurements of Total and Regional Body Composition in Preschool Children: A Comparison of MRI, DXA, and Anthropometric Data. Obesity 21 (5), 1018–1024. 10.1002/oby.20205 23784906

[B24] Khandekar M. J. Cohen P. Spiegelman B. M. (2011). Molecular Mechanisms of Cancer Development in Obesity. Nat. Rev. Cancer 11 (12), 886–895. 10.1038/nrc3174 22113164

[B25] Leone P. Shin E.-C. Perosa F. Vacca A. Dammacco F. Racanelli V. (2013). MHC Class I Antigen Processing and Presenting Machinery: Organization, Function, and Defects in Tumor Cells. JNCI J. Natl. Cancer Inst. 105 (16), 1172–1187. 10.1093/jnci/djt184 23852952

[B26] Liedtke S. Schmidt M. E. Vrieling A. Lukanova A. Becker S. Kaaks R. (2012). Postmenopausal Sex Hormones in Relation to Body Fat Distribution. Obesity (Silver Spring, Md) 20 (5), 1088–1095. 10.1038/oby.2011.383 22240723

[B27] Ling C. Rönn T. (2019). Epigenetics in Human Obesity and Type 2 Diabetes. Cel Metab. 29 (5), 1028–1044. 10.1016/j.cmet.2019.03.009 PMC6509280 30982733

[B28] Lyman G. H. (2012). Weight-based Chemotherapy Dosing in Obese Patients with Cancer: Back to the Future. Jop 8 (4), e62–e64. 10.1200/jop.2012.000606 23181001 PMC3396832

[B29] Maguire O. A. Ackerman S. E. Szwed S. K. Maganti A. V. Marchildon F. Huang X. (2021). Creatine-mediated Crosstalk between Adipocytes and Cancer Cells Regulates Obesity-Driven Breast Cancer. Cel Metab. 33 (3), 499–512. e496. 10.1016/j.cmet.2021.01.018 PMC7954401 33596409

[B30] McGettrick A. F. O’Neill L. A. J. (2020). The Role of HIF in Immunity and Inflammation. Cel Metab. 32 (4), 524–536. 10.1016/j.cmet.2020.08.002 32853548

[B31] Neamat-Allah J. Wald D. Hüsing A. Teucher B. Wendt A. Delorme S. (2014). Validation of Anthropometric Indices of Adiposity against Whole-Body Magnetic Resonance Imaging - A Study within the German European Prospective Investigation into Cancer and Nutrition (EPIC) Cohorts. PloS one 9 (3), e91586. 10.1371/journal.pone.0091586 24626110 PMC3953447

[B32] Nimptsch K. Konigorski S. Pischon T. (2019). Diagnosis of Obesity and Use of Obesity Biomarkers in Science and Clinical Medicine. Metabolism 92, 61–70. 10.1016/j.metabol.2018.12.006 30586573

[B33] Picon-Ruiz M. Morata-Tarifa C. Valle-Goffin J. J. Friedman E. R. Slingerland J. M. (2017). Obesity and Adverse Breast Cancer Risk and Outcome: Mechanistic Insights and Strategies for Intervention. CA: a Cancer J. clinicians 67 (5), 378–397. 10.3322/caac.21405 PMC5591063 28763097

[B34] Pierobon M. Frankenfeld C. L. (2013). Obesity as a Risk Factor for Triple-Negative Breast Cancers: a Systematic Review and Meta-Analysis. Breast Cancer Res. Treat. 137 (1), 307–314. 10.1007/s10549-012-2339-3 23179600

[B35] Prentice A. M. Jebb S. A. (2001). Beyond Body Mass index. Obes. Rev. 2 (3), 141–147. 10.1046/j.1467-789x.2001.00031.x 12120099

[B36] Quail D. F. Dannenberg A. J. (2019). The Obese Adipose Tissue Microenvironment in Cancer Development and Progression. Nat. Rev. Endocrinol. 15 (3), 139–154. 10.1038/s41574-018-0126-x 30459447 PMC6374176

[B37] Ritchie M. E. Phipson B. Wu D. Hu Y. Law C. W. Shi W. (2015). Limma powers Differential Expression Analyses for RNA-Sequencing and Microarray Studies. Nucleic Acids Res. 43 (7), e47. 10.1093/nar/gkv007 25605792 PMC4402510

[B38] Samblas M. Milagro F. I. Martínez A. (2019). DNA Methylation Markers in Obesity, Metabolic Syndrome, and Weight Loss. Epigenetics 14 (5), 421–444. 10.1080/15592294.2019.1595297 30915894 PMC6557553

[B39] Shah S. P. Roth A. Goya R. Oloumi A. Ha G. Zhao Y. (2012). The Clonal and Mutational Evolution Spectrum of Primary Triple-Negative Breast Cancers. Nature 486 (7403), 395–399. 10.1038/nature10933 22495314 PMC3863681

[B40] Sung H. Siegel R. L. Torre L. A. Pearson-Stuttard J. Islami F. Fedewa S. A. (2019). Global Patterns in Excess Body Weight and the Associated Cancer burden. CA Cancer J. Clin. 69 (2), 88–112. 10.3322/caac.21499 30548482

[B41] Suzuki R. Orsini N. Saji S. Key T. J. Wolk A. (2009). Body Weight and Incidence of Breast Cancer Defined by Estrogen and Progesterone Receptor Status-A Meta-Analysis. Int. J. Cancer 124 (3), 698–712. 10.1002/ijc.23943 18988226

[B42] Taylor C. T. Colgan S. P. (2017). Regulation of Immunity and Inflammation by Hypoxia in Immunological Niches. Nat. Rev. Immunol. 17 (12), 774–785. 10.1038/nri.2017.103 28972206 PMC5799081

[B43] Teschendorff A. E. Gao Y. Jones A. Ruebner M. Beckmann M. W. Wachter D. L. (2016). DNA Methylation Outliers in normal Breast Tissue Identify Field Defects that Are Enriched in Cancer. Nat. Commun. 7, 10478. 10.1038/ncomms10478 26823093 PMC4740178

[B44] Tian Y. Morris T. J. Webster A. P. Yang Z. Beck S. Feber A. (2017). ChAMP: Updated Methylation Analysis Pipeline for Illumina BeadChips. Bioinformatics 33 (24), 3982–3984. 10.1093/bioinformatics/btx513 28961746 PMC5860089

[B45] von Boehmer H. Daniel C. (2013). Therapeutic Opportunities for Manipulating TReg Cells in Autoimmunity and Cancer. Nat. Rev. Drug Discov. 12 (1), 51–63. 10.1038/nrd3683 23274471

[B46] Wahl S. Drong A. Lehne B. Loh M. Scott W. R. Kunze S. (2017). Epigenome-wide Association Study of Body Mass index, and the Adverse Outcomes of Adiposity. Nature 541 (7635), 81–86. 10.1038/nature20784 28002404 PMC5570525

[B47] Wang Z. Aguilar E. G. Luna J. I. Dunai C. Khuat L. T. Le C. T. (2019). Paradoxical Effects of Obesity on T Cell Function during Tumor Progression and PD-1 Checkpoint Blockade. Nat. Med. 25 (1), 141–151. 10.1038/s41591-018-0221-5 30420753 PMC6324991

[B48] Yoshihara K. Shahmoradgoli M. Martínez E. Vegesna R. Kim H. Torres-Garcia W. (2013). Inferring Tumour Purity and Stromal and Immune Cell Admixture from Expression Data. Nat. Commun. 4, 2612. 10.1038/ncomms3612 24113773 PMC3826632

[B49] Zhang C. Yue C. Herrmann A. Song J. Egelston C. Wang T. (2020). STAT3 Activation-Induced Fatty Acid Oxidation in CD8+ T Effector Cells Is Critical for Obesity-Promoted Breast Tumor Growth. Cel Metab. 31 (1), 148–161. e145. 10.1016/j.cmet.2019.10.013 PMC6949402 31761565

