# Supplementary figures and images for: Integrative Analysis of Gene Expression and DNA Methylation Depicting the Impact of Obesity on Breast Cancer
Source: Front Cell Dev Biol. 2022 Mar 8;10:818082. doi: 10.3389/fcell.2022.818082 (PMC8957964; doi:10.3389/fcell.2022.818082)

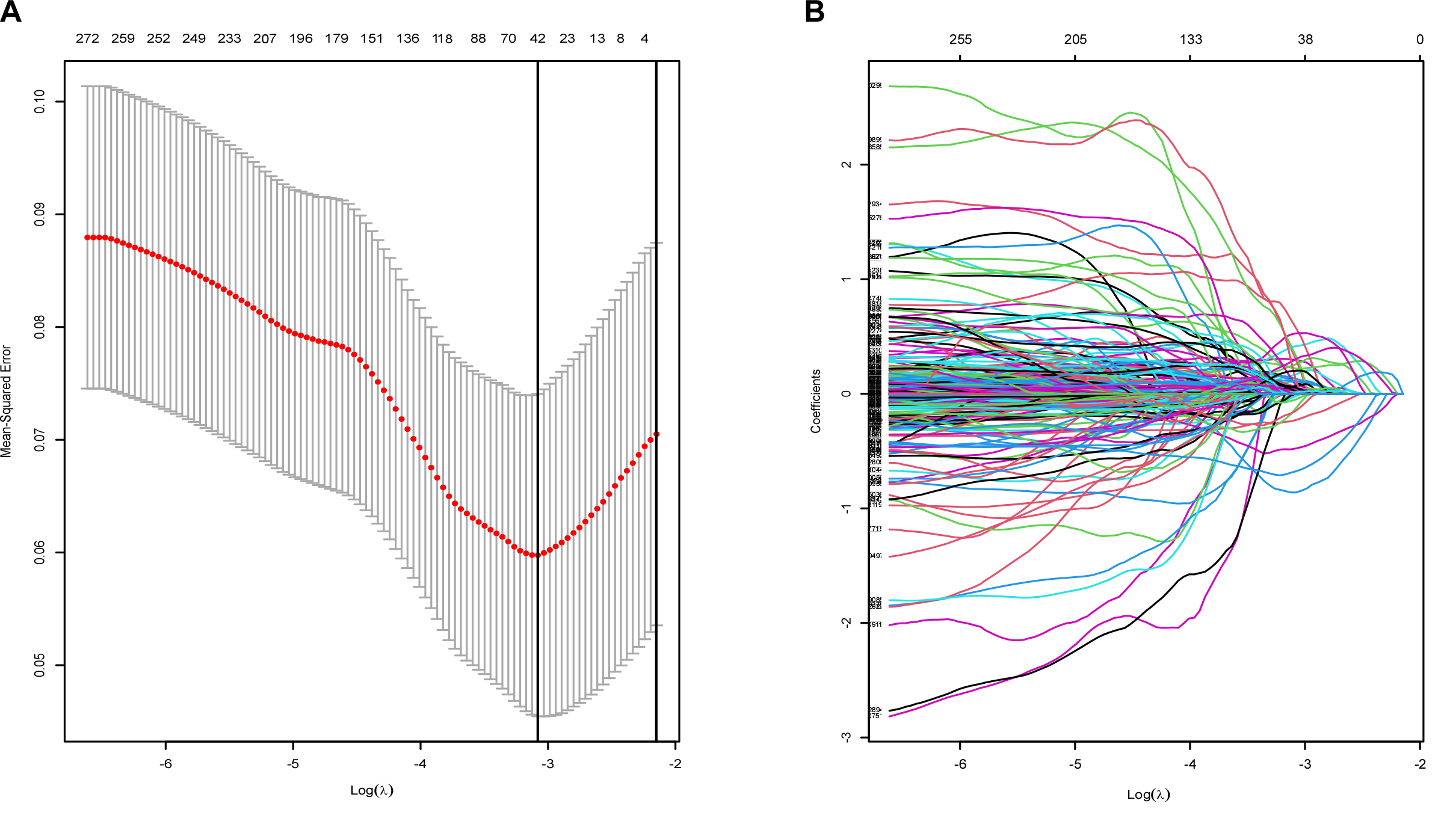

Supplement: Supplementary file 6 [file Image1.TIF]
